# Seasonal Patterns of Fine Root Production and Turnover in a Mature Rubber Tree (*Hevea brasiliensis* Müll. Arg.) Stand- Differentiation with Soil Depth and Implications for Soil Carbon Stocks

**DOI:** 10.3389/fpls.2015.01022

**Published:** 2015-11-27

**Authors:** Jean-Luc Maeght, Santimaitree Gonkhamdee, Corentin Clément, Supat Isarangkool Na Ayutthaya, Alexia Stokes, Alain Pierret

**Affiliations:** ^1^Institut de Recherche pour le Développement, UMR 242/iEES – Paris (IRD-UPMC-CNRS-UPEC-UDD-INRA)Bondy, France; ^2^INRA, UMR-AMAPMontpellier, France; ^3^Khon Kaen University, Faculty of AgricultureKhon Kaen, Thailand; ^4^International Water Management InstituteVientiane, Laos; ^5^Institut de Recherche Pour le Développement, UMR IEES-Paris – Department of Agricultural Land Management (DALaM)Vientiane, Laos

**Keywords:** deep roots, root phenology, root turnover, soil carbon, root access well, drought

## Abstract

Fine root dynamics is a main driver of soil carbon stocks, particularly in tropical forests, yet major uncertainties still surround estimates of fine root production and turnover. This lack of knowledge is largely due to the fact that studying root dynamics *in situ*, particularly deep in the soil, remains highly challenging. We explored the interactions between fine root dynamics, soil depth, and rainfall in mature rubber trees (*Hevea brasiliensis* Müll. Arg.) exposed to sub-optimal edaphic and climatic conditions. A root observation access well was installed in northern Thailand to monitor root dynamics along a 4.5 m deep soil profile. Image-based measurements of root elongation and lifespan of individual roots were carried out at monthly intervals over 3 years. Soil depth was found to have a significant effect on root turnover. Surprisingly, root turnover increased with soil depth and root half-life was 16, 6–8, and only 4 months at 0.5, 1.0, 2.5, and 3.0 m deep, respectively (with the exception of roots at 4.5 m which had a half-life similar to that found between depths of 1.0 and 2.5 m). Within the first two meters of the soil profile, the highest rates of root emergence occurred about 3 months after the onset of the rainy season, while deeper in the soil, root emergence was not linked to the rainfall pattern. Root emergence was limited during leaf flushing (between March and May), particularly within the first two meters of the profile. Between soil depths of 0.5 and 2.0 m, root mortality appeared independent of variations in root emergence, but below 2.0 m, peaks in root emergence and death were synchronized. Shallow parts of the root system were more responsive to rainfall than their deeper counterparts. Increased root emergence in deep soil toward the onset of the dry season could correspond to a drought acclimation mechanism, with the relative importance of deep water capture increasing once rainfall ceased. The considerable soil depth regularly explored by fine roots, even though significantly less than in surface layers in terms of root length density and biomass, will impact strongly the evaluation of soil carbon stocks.

## Introduction

Fine root production and turnover represent 22% of terrestrial net primary production globally (McCormack et al., 2015). Yet there are still major uncertainties about the mechanisms that control fine root production and turnover. With the growing global demand for food and plant-derived commodities, unraveling such mechanisms is becoming increasingly important, particularly with the concomitantly pressing need to develop more sustainable agro-ecosystems. In recent years, rubber tree (*Hevea brasiliensis* Müll. Arg.) plantations have rapidly expanded, especially in marginal regions where the climate is much drier than in the species’ natural range and where seasonal drought occurs (Carr, 2011). Soaring prices of natural rubber in the late 2010s influenced governmental policies regarding the expansion of *H. brasiliensis* cultivation. In Thailand, the world’s leading latex producer, where the surface area planted with *H. brasiliensis* was multiplied by a factor of 75 between 1980 and 2008, from 24,000 ha^-1^ to 1.8 million ha^-1^ (Carr, 2011). Given the stress that tapping, i.e., the process by which the latex is collected, already imposes on *H. brasiliensis*, the sustainability and profitability of latex production in such areas could greatly benefit from adapting tapping modalities by taking into account the physiological response of trees to water availability (Boithias et al., 2011; Junjittakarn, 2012).

As roots are conduits for nutrients and water from the soil to plants, they have a determining role with regard to tree resilience to a range of environmental constraints, especially water stress (Boyce, 2005; Lobet et al., 2013). Fine roots are also an integrative indicator of plant response to environmental factors (Edwards et al., 2004) and we assume that root production or elongation of *H. brasiliensis* is synchronized with rainfall patterns (Green et al., 2005), although there exists evidence of endogenous controls of root growth (Abramoff and Finzi, 2015). Therefore, we expect that fine root growth is arrested during the dry season, but no data exist to support or refute this hypothesis. Fine roots of trees contribute to soil water extraction (Danjon et al., 2013), while a variable (and most often poorly quantified) share of the water demand is supplied by deep roots (Maeght et al., 2013). The measurement of root growth and survival *in situ* along a deep soil profile is an approach that can bring essential information to understanding how trees cope with water-limiting conditions and tree resilience to such constraints.

Quantifying fine root phenology and mortality down a soil profile, and particularly in deep soils, will also impact the evaluation of belowground carbon stocks, an area where data are scarce (Wauters et al., 2008). This proportion of soil carbon stocks could well contribute to the balance between the release and accumulation of carbon fluxes, currently described as the “missing sink” (Esser et al., 2011). However, to observe and analyze roots non-destructively within the soil matrix is still a major scientific challenge (Virginia and Jarrell, 1987), especially in deep soil layers and most studies have focused on the superficial layers of soil (Stone and Kalisz, 1991; Maeght et al., 2013). Our knowledge of fine root lifespan is also limited, particularly at depth (Eissenstat and Yanai, 1997).

We hypothesize that: (i) rooting in general is deeper than commonly assumed, (ii) fine root phenology and mortality are synchronized with annual patterns of precipitation, (iii) fine roots growing deep in the soil contribute to the resilience of *H. brasiliensis* to frequently occurring drought conditions in N. E. Thailand. Therefore, we measured seasonal patterns of fine root production and turnover in a mature stand of *H. brasiliensis*, down a 4.5 m soil profile during a 3-year observation period. We examined the interactions between fine root dynamics, rainfall, and soil depth and estimated the relative contribution of fine and deep roots to soil carbon.

## Materials and Methods

### Study Site and Climate

The field site was located at Baan Sila Khu-Muang village, Buriram province in North East Thailand (N 15°16′23″, E 103°04′51.3″, 150 m a.s.l.). This region is part of the non-traditional areas for *H. brasiliensis* cultivation established since the 1990s. The experiment was set up in 2006 in a monoclonal plot of 14 years old trees (RRIM 600 clone developed by the Rubber Research Institute of Malaysia), planted at 2.5 m × 7.0 m spacing (∼570 trees ha^-1^) that had already been tapped for over 4 years to produce latex. Tapping was performed using a semi-spiral cut 2 days out of 3 and is largely adapted to the local climatic conditions. Tapping is usually discontinued for 3–4 months during the dry season. The maximum leaf area index (LAI), measured using 91 m^1^ litter traps during the defoliation period (December–February; Isarangkool Na Ayutthaya et al., 2010), was estimated to be 3.9 ± 0.7 (mean ± standard deviation).

This marginal area for rubber tree cultivation is subjected to the Southeast Asian monsoon, with heavy rainfall between April and October. Local microclimate was monitored automatically with a Minimet weather station (Skye Instruments Ltd, UK) attached to a data logger recording air temperature, relative humidity, incident short wave radiation and rainfall at 30-min intervals. Reference evapotranspiration (ET0) was calculated according to Allen et al. (1998) using the data collected from the weather station.

### Soil Properties

The soil at the study site was a deep loamy sand with limited water retention capacity, developed on fine sand or coarse silt deposits with a homogeneous sandy loam texture throughout the profile. The Ap horizon was a 0.25 m thick remnant from previous sugar cane (*Sacharum officinarum L.*) cultivation (Hartmann et al., 2006). Clay, silt, and sand contents were 100, 100, and 800 g kg^-1^, respectively. The clay content increased with depth: from 150 g kg^-1^ in the Bt1 horizon (0.25–0.50 m) to 200 g kg^-1^ in the Bt2 (0.50–1.0 m). Silt content was similar in all soil layers throughout the soil profile (100 g kg^-1^) while sand decreased to 700 g kg^-1^ at a depth of 1.0 m. From 100 to about 4.0 m, these properties remained fairly stable. The laterite layer was found at a depth of 6.0–7.0 m, as previously observed in this region (Cawte and Boyd, 2010). The water table was not found within the first 7 m of the profile, even during the rainy season. The soil was acidic with a pH ranging from 5.0 to 5.3. Soil organic carbon content was lower than 10 g kg^-1^ in the topsoil (Isarangkool Na Ayutthaya et al., 2011). Typical bulk soil density was 1.55 g cm^-3^ to a depth of 3.0 m (Gonkhamdee et al., 2009). Additional soil properties can be found in Hartmann et al. (2006) and Isarangkool Na Ayutthaya et al. (2011).

### Root Growth Monitoring and Rooting Profiles

#### Soil Coring

To quantify carbon stocks associated with fine roots, root samples were collected at depths corresponding to the depths covered by root windows. We extracted undisturbed soil cores using standard soil sample steel rings (diameter 53 mm, height 50 mm and 100 cm^3^ internal volume, Eijkelkamp Giesbeek, The Netherlands), in the vicinity of the root access well (*n* = 12 at soil depths 0.25, 0.50, 0.75, and 1.0 m; *n* = 5 at soil depths 1.6, 2.8, and 4.0 m). Root samples were analyzed according to Pierret et al. (2007b). Roots were first washed free of soil from the undisturbed soil cores and then imaged using a flatbed scanner (Epson Perfection V700 Photo scanner; Seiko Epson Corp., Japan) in light transmission mode, at a spatial resolution of 600 dpi (pixel size of 0.0423 mm). Special care was taken to separate every root from each other as much as possible, since overlapping roots are known to impair accurate length recovery. Specific root length (SRL) values, i.e., the length of root per unit dry root biomass, obtained from fine root samples collected within the first meter of the soil profile, were used to estimate the root dry biomass (RDB) distribution along the 4.5 m profile observed in the well, based on the following equation:

(1) RDB=RLD×[Z]/SRL⁢

where RDB (in Mg ha^-1^) is the RDB in a soil depth layer of thickness [Z] (m), RLD is the root length density (m m^-3^) calculated from soil cores, in this soil layer and SRL the specific root length (m g^-1^).

### Root Access Well

Root growth was studied using the access well technique described in Maeght et al. (2013). An access well (0.9 m in diameter and 4.5 m deep) was installed in July 2006 at a distance of 1.35 m from two trees and 0.5 m aside from a tree row. The access well observation technique is an evolution of basic techniques for root observation at transparent interfaces with soil (Smit et al., 2000). A total of nine observation windows were cut through the concrete walls of the well in staggered rows (with 1.0 and 0.5 m horizontal and vertical spacing, respectively). Each root window included a specifically designed metallic frame supporting, on its upper side, a piece of 8 mm thick glass (0.25 m × 0.30 m) pressed against the soil at a 45° angle by means of two threaded rod actuators. On the frame’s lower side, two guide rails allow the insertion of a standard flatbed scanner. Overall, given the geometrical arrangement of windows, the soil depth increments that were accessible were 0.4–0.6, 0.9–1.1, 1.4–1.6, 1.9–2.1, 2.4–2.6, 2.9–3.1, 3.4–3.6, 3.9–4.1, and 4.4–4.6 m. For simplicity, these are referred to as 0.5, 1.0, 1.5, 2.0, 2.5, 3.0, 3.5, 4.0, and 4.5 m hereafter. Due to time and financial constraints, it was not possible to build and monitor replicate root access wells within the framework of this field experiment. More details about the set up of the root access well set be found in Gonkhamdee et al. (2009) and Maeght et al. (2013).

Images of roots were taken using a flatbed scanner (HP Scanjet 4370 Photo scanner at 200 DPI – Hewlett-Packard Development Company, California) and custom software which offers a convenient, faster and more accurate record than manual techniques (Zoon and Tienderen, 1990; Kaspar and Ewing, 1997). Root windows were scanned at monthly intervals during 3 years starting in January 2007. This scanning was started 6 months after setting up the access well to avoid recording overproduction of roots at the onset, as often occurs in mini-rhizotron experiments (Yuan and Chen, 2012). Long-term observations are also highly recommended to avoid the risk of overestimating fine root turnover (Strand et al., 2008). Root growth monitoring was conducted following a procedure described in Maeght et al. (2007). Images of the soil and roots in direct contact with each window were used to estimate root length, radius, and time of root appearance/disappearance (from which root turnover was inferred). We used the Gimp freeware package^1^ to digitize roots and classify them as live or senescent. Senescent roots are often difficult to identify with certainty (Majdi et al., 2005). We considered roots as senescent when they exhibited no elongation and/or radius expansion for at least two successive observation dates and when their color turned from white to brown. Senescent roots were considered dead when their color changed to dark brown/black or when they completely disappeared from one observation to the next. A total of more than 1500 roots distributed in 300 images were processed.

Root emergence was quantified as the number of roots appearing between two monthly observations divided by the number of root windows from which this number of roots was derived (number of new roots per cm^2^ and per month). Likewise, root mortality was quantified as the number of roots that disappeared between two monthly observations divided by the number of root windows from which this number of roots was derived (number of senescent/dead roots per root window and per month). 95% confidence intervals were computed as an indicator of the variability of root emergence/mortality across windows.

Assuming that all roots had emerged at the same time, the half-life represents the time after which half of all the roots would have died. Half-life values simplify comparisons between the survival of roots that emerged at the onset of the observation period and for which the time of disappearance could actually be recorded and that of roots that emerged much later and which died after the end of the observation period (right-censored data).

### Analysis of Root Sample Images

Root length measurements were obtained using IJ_Rhizo’s implementation of the method developed by Kimura et al. (1999). IJ_Rhizo (Pierret et al., 2013) is a software designed to measure roots washed from soil samples and developed in the ImageJ^2^ macro language. The approach developed by Kimura et al. (1999) is based on discriminating each pixel of the medial axis (or skeleton) of each digitized root according to its number of orthogonal and diagonal (vertical or horizontal) neighbors. We also used IJ_Rhizo to compute frequency distributions of root radius classes (i.e., the cumulated root length par root radius class).

### Statistical Analyses

All numerical data processing and statistical analyses were performed within the R environment (version 3.0.2; R Development Core Team, 2013). We first explored our dataset using a principal component analysis (PCA; “ade4” package, version 1.6-2). RLD and root radius values are reported as mean ± 95% confidence interval. We applied analysis of variance with Tukey’s HSD *post hoc* tests to determine differences in root radius at different soil depths. We assessed fine root survival at the depth of each root using a Kaplan–Meier survival analysis (Kaplan and Meier, 1958) implemented in the “*survival*” package (version 2.37-4) of the R environment. For each individual root, we recorded the time of emergence and the time to either an event (death) or the end of the study (i.e., roots that were still alive at the time of the last observation were right-censored). Differences in survivorship of roots that emerged at different soil depths (regardless of their actual time of emergence) were assessed by *post hoc* pairwise comparisons using the Mann–Whitney test with a Bonferroni correction. Additionally, a Cox proportional hazards regression model was used to test whether root radius had an influence on root survival. Fine root emergence and mortality determined for each root observation window at monthly intervals are reported as pseudo-medians derived from the Wilcoxon test ± 95% confidence intervals.

A PCA (Supplementary Figure S1) indicated that root emergence was partly explained by rainfall and evapotranspiration, but the two first axes accounted for less than 50% of the variance. Therefore, we resorted here to a more descriptive analysis of fine root dynamics. As (i) roots with lifespans of 30 months and more only occurred between the surface and a depth of 2.0 m, (ii) roots were thicker above 2.0 m (with the exception of the 1.5 m depth increment) and (iii) at depths below 2.0 m, roots emerged at least 12 months later than at depths above 2.0 m, we chose to analyze separately root dynamics above and below the soil depth of 2.0 m.

## Results

### Climate Measurements

**FIGURE 1:**
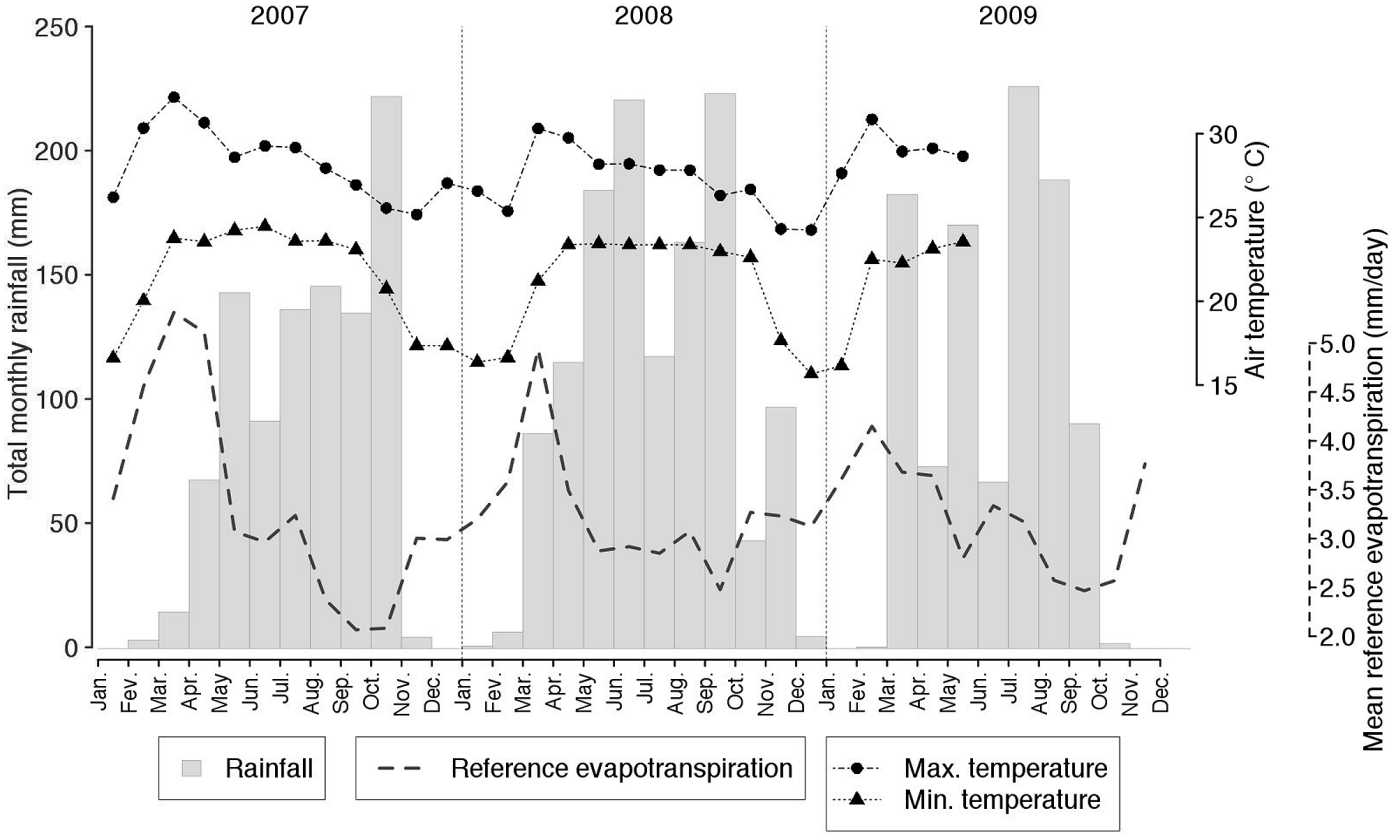
**Monthly rainfall, minimum and maximum air temperatures and reference evapotranspiration monitored at the experimental site from January 2007 to December 2009**.

Total annual rainfall during the 3-year period over which the experiment was conducted, was 965, 1265, and 1002 mm, in 2007, 2008, and 2009, respectively (average: 1077 mm; **Figure 1**). Air temperatures increased during the dry season and decreased following the end of the rainy season (with a range from +8.3 to +40.3°C). Reference evapotranspiration (ET0) was found to roughly follow the monsoon regime, with a peak toward the end of the dry season and a subsequent decrease throughout the rainy season (**Figure 1**).

### Rooting Profiles

**FIGURE 2:**
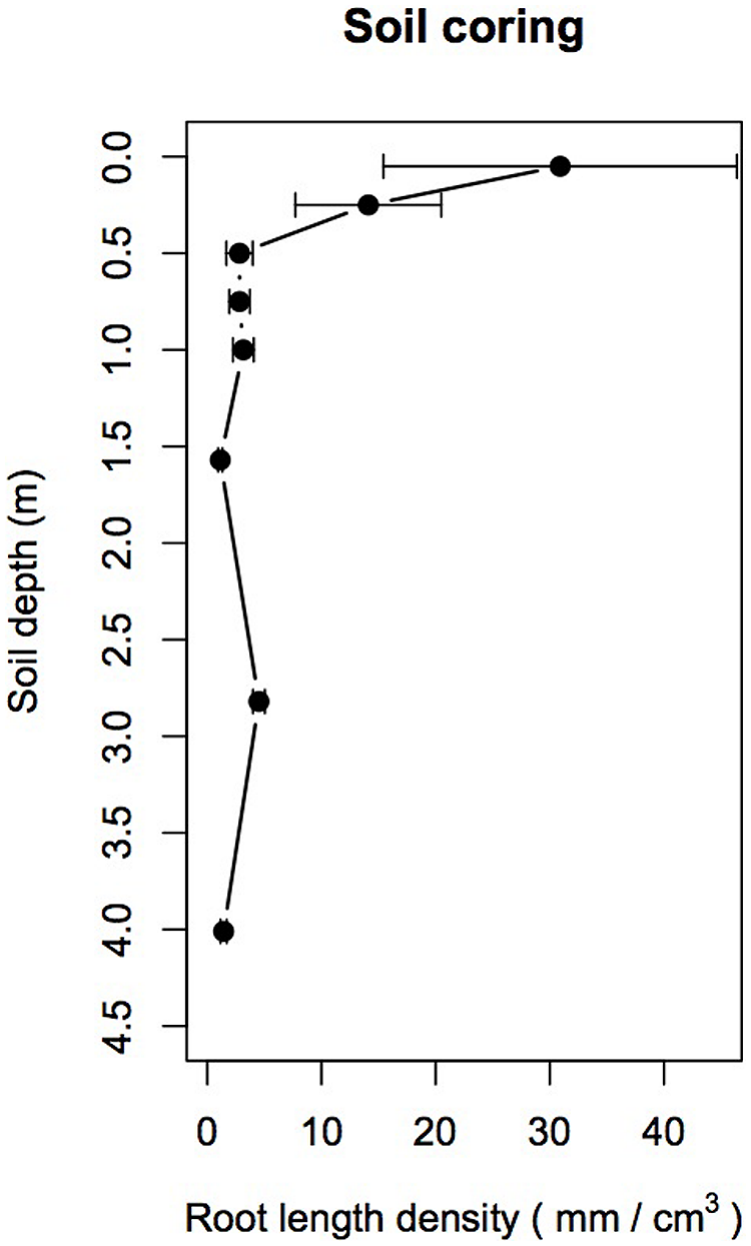
**Mean root length density (RLD mm/cm^3^) derived from soil coring (*n* = 12 per depth between 0.05 and 1.0 m; *n* = 5 below 1.0 m) as a function of soil depth.** Note the significant decrease of RLD with increasing soil depth below 0.25 m and the relative increase of RLD between 2.5 and 3.0 m. Dots are mean values and error bars are 95% confidence intervals.

Mean fine RLD derived from soil coring decreased by about one order of magnitude from a depth of 0.05 to 0.5 m, then declined slightly from 0.5 to 1.5 m before further increasing at 2.82 m (*F*_7,55_ = 7.49, *p* < 0.001; **Figure 2**). A *post hoc* Tukey test showed that mean fine RLD was significantly higher at 0.05 m than at all other depths (*p* < 0.05) and that fine root RLD between 0.25 and 4.0 m were not significantly different from each other.

**FIGURE 3:**
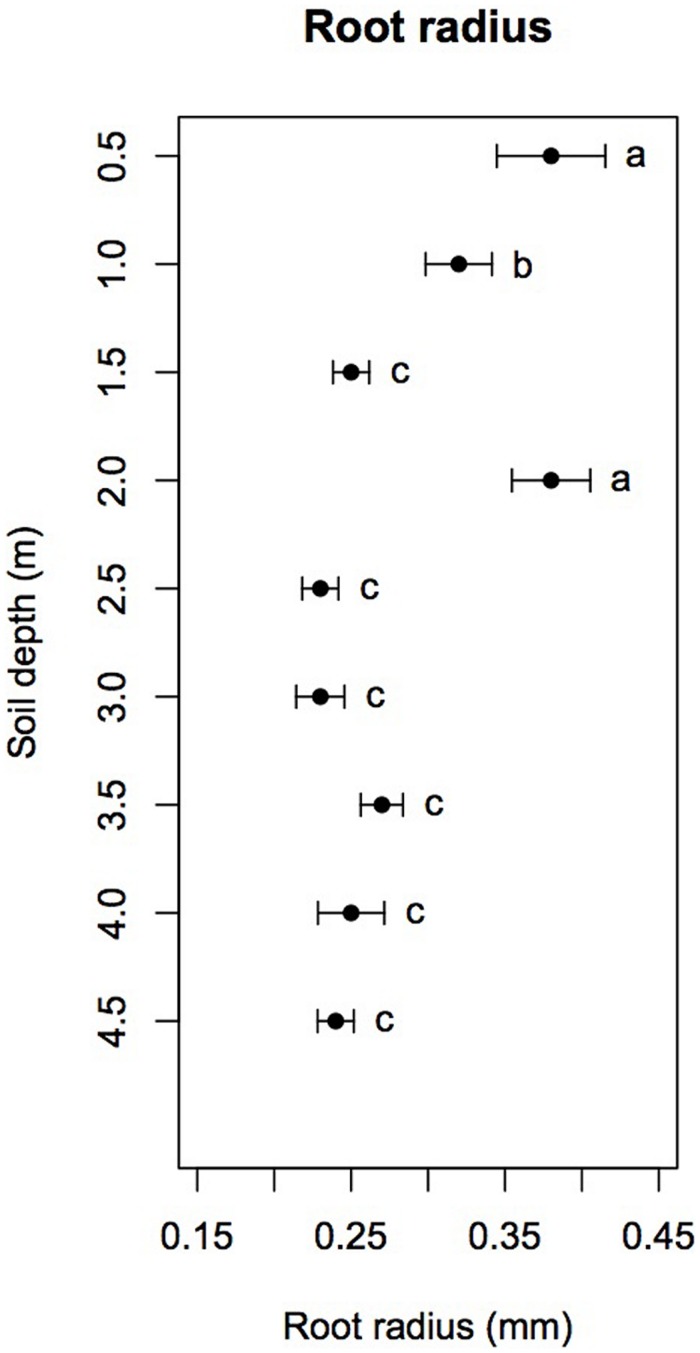
**Mean root radius as a function of soil depth (dots) with 95% confidence intervals (error bars).** Different letters indicate significant differences (Tukey HSD with α = 0.05).

Mean root radius measured in root windows significantly varied with soil depth (*F*_8,89_ = 34.15, *p* < 0.001), reaching 0.38 ± 0.03 mm at 0.5, 0.38 ± 0.02 mm at 2.0 m and 0.32 ± 0.02 mm at 1.0 m. At all other soil depths, mean root radius was fairly constant at 0.23–0.27 mm (**Figure 3**). A *post hoc* Tukey HSD test showed that mean root radii at 0.5 and 2.0 m were significantly higher than those at 1.0 m at *p* < 0.05 and that mean root radius at 1.0 m was itself significantly higher than those at all other soil depths (*p* < 0.05).

### Root Emergence and Age Distributions

**FIGURE 4:**
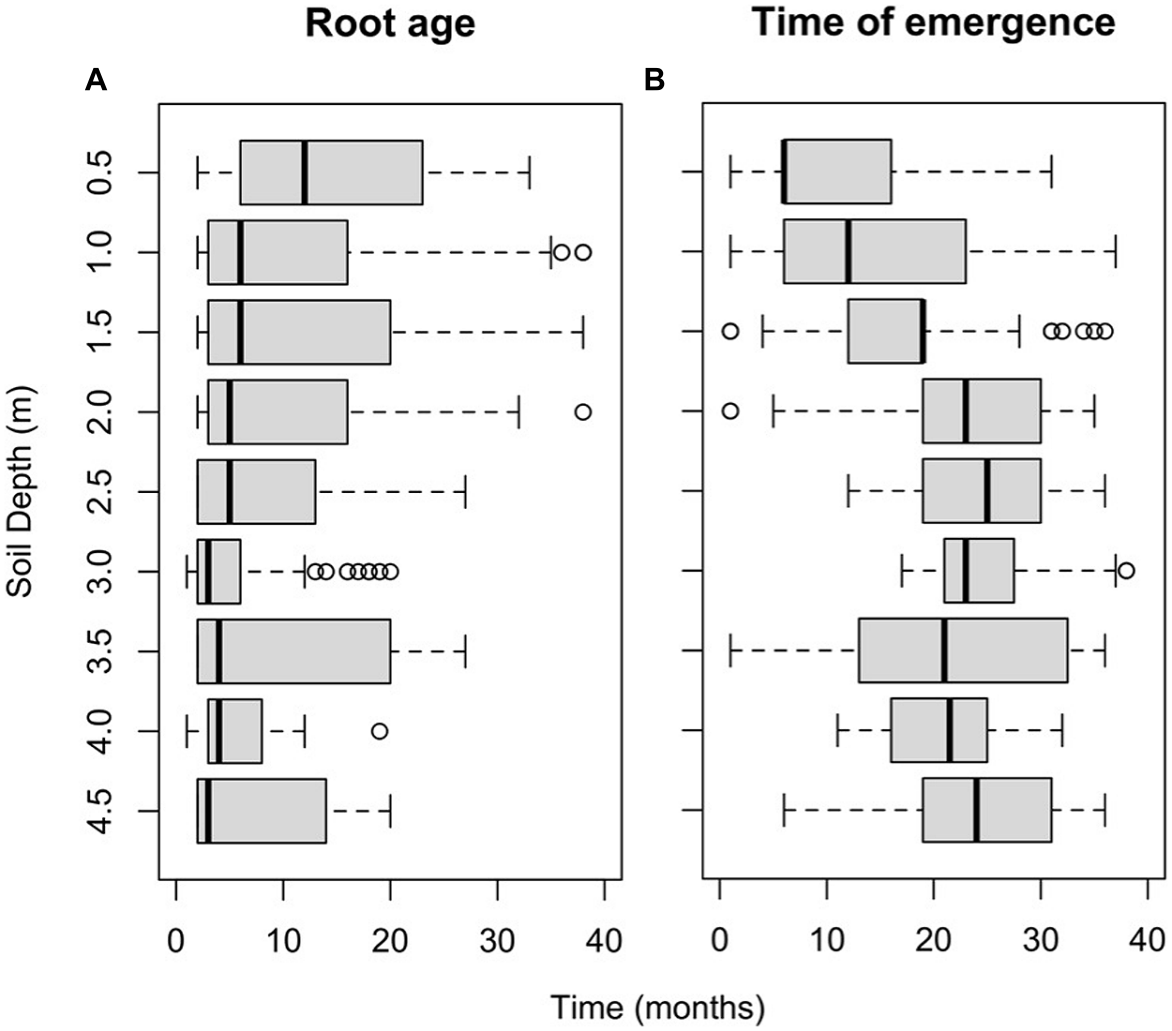
**(A)** Box-whisker plot of root age distributions in each root window (for live and dead roots combined). **(B)** Distribution of the times of emergence of roots for each root window. The central vertical line indicates the median value, and the left and right edges of boxes (hinges) correspond to the 25th and 75th percentile values, while the whiskers extend 1.5× beyond the spread of the hinges. Data points outside this range (outliers) are indicated with circles.

Roots near the soil surface (0.0–0.5 m) had a significantly longer life span compared to that in deeper soil layers (**Figure 4A**). There was a significant effect of soil depth on root age (*c*^2^ = 94.93, *p* < 0.001). Roots with life spans of 30 months and more were only observed between the surface and a depth of 2.0 m.

Roots first emerged in layers close to the soil surface (0.5 and 1.0 m) and at 3.5 m, i.e., within the first 6 months of the observation period. However, at depths of 2.5, 3.0, and 4.0 m, roots did not emerge until 11–17 months after the beginning of the observation period (**Figure 4B**). Root emergence differed significantly depending on soil depth (*F*_8,89_ = 34.15, *p* < 0.001). Root emergence occurred significantly earlier (*p* < 0.05) in the three top windows (means were: 11, 14, and 17 months at 0.5, 1.0, and 1.5 m, respectively) than in the deeper windows (means were: 21–25 months).

### Root Survival

**FIGURE 5:**
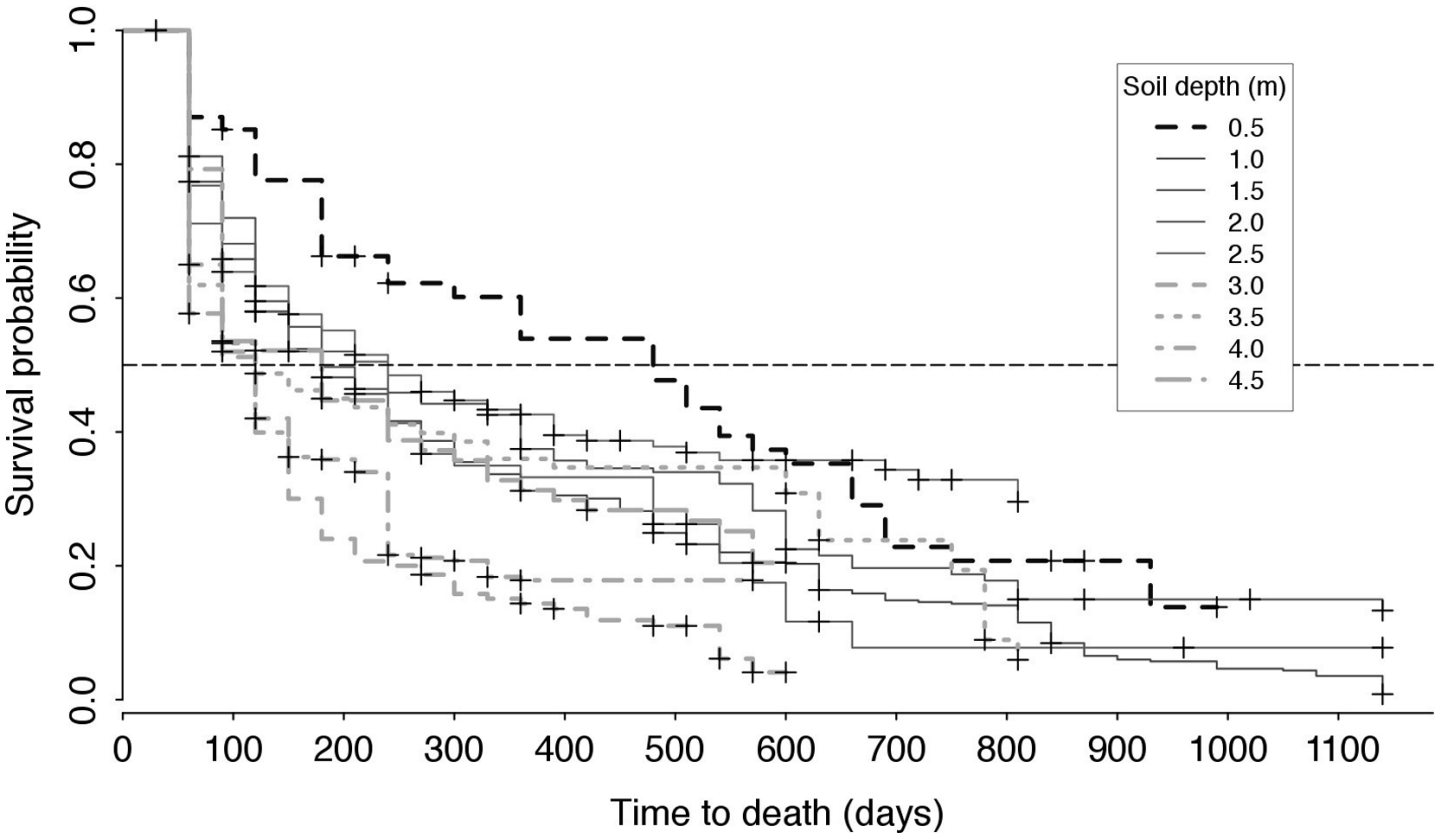
**Kaplan–Meier survival probability of roots depending on soil depth (see legend).** The dotted horizontal line indicates a survival probability of 50%: the abscissa at which a given Kaplan–Meier curves intersects this line represents the half-life of roots corresponding to this survival curve, i.e., the estimated time required for half of the roots observed at any given point in time to have died.

Kaplan and Meier (1958) curves showed that, overall, root half-life decreased with soil depth, with the half-life of roots at 0.5 m being in the order of 500 days (>16 months; turnover of 0.73 yr^-1^). The half-life of roots between 1.0 and 2.5 m was about 180–250 days (6–8 months; turnover 1.46–2.03 yr^-1^) and that of roots at 3.0 m and below dropped to less than 120 days (∼4 months; turnover 3.04 yr^-1^), with the exception of roots at 4.5 m; the latter had a half-life similar to that found between 1.0 and 2.5 m (**Figure 5**). Soil depth significantly affected root half-life values (χ^2^ = 89.9, *p* < 0.001) and survival at 0.5 m was longer than that at all other depths (*p* < 0.05) except for 2.5 and 1.5 m, while there was no difference in root survival between depths of 3.5–4.5 m.

Differences in root survival might be related in part, to root branching order, with higher branching order roots (Pregitzer et al., 2002), living longer, i.e., thicker roots observed at 0.5 m (**Figure 3**). This could be putatively associated with slower turnover compared to lower order roots (Yao et al., 2009; Sun et al., 2012). However, a Cox proportional hazards model including root radius as a covariate of soil depth showed that root survival was clearly influenced by soil depth (*p* < 0.001) and not by root radius (*p* = 0.25).

### Root Dynamics as a Function of Soil Depth and Rainfall

**FIGURE 6:**
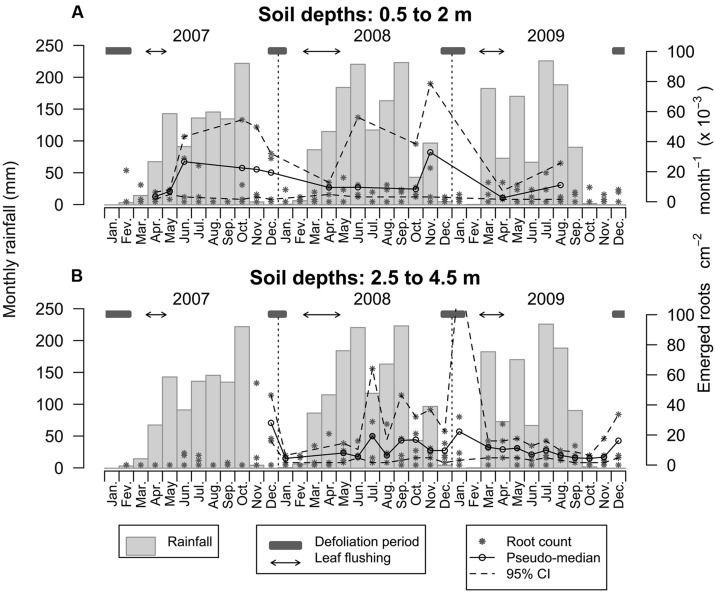
**Dynamics of root emergence over 3 years within the top 0.5–2.0 m of the soil profile (A) and between 2.5 and 4.5 m (B).** Asterisks are emerging root counts determined in every root window at monthly intervals. Open circles represent the pseudo-median of emerging root counts in root windows (*n* = 4 and *n* = 5 for the upper and lower plots, respectively) and dashed lines are 95% confidence intervals estimated using the Wilcoxon signed rank test.

Root emergence between soil depths of 0.5 and 2.0 m ranged from 1.60 to 107.42 × 10^-3^ emerging roots cm^-2^ month^-1^, with an average of 7.33 × 10^-3^ emerging roots cm^-2^ month^-1^ (**Figure 6A**). Despite much variability, root emergence tended to be lowest in the first 3–4 months of each observed year, followed by an increase that lasted at least until the month of November (although there was much variability between depth increments and observation years, **Figure 6A**). During the first 2 years, root emergence increased approximately 3 months after the first rainfall, usually in the month of June. Root emergence could occur at relatively high rates during defoliation but was generally low during leaf flushing (**Figure 6A**). The dynamics of root emergence observed below 2.0 m was radically different with root emergence ranging from 1.60 to 128.27 × 10^-3^ emerging roots cm^-2^ month^-1^, with a mean of 7.11 × 10^-3^ emerging roots cm^-2^ month^-1^ (**Figure 6B**). There was very limited root growth until the 11th month of the observation period – or 14 months after root windows were installed – (i.e., until the onset of the first dry season and during leaf fall). Beyond that point in time, root emergence subsided until February 2008 and increased again and remained relatively high for 1 year (with a mean of 11.81 × 10^-3^ emerging roots cm^-2^ month^-1^). Subsequently, root emergence slowed down and became more stable over time with a mean of 6.57 × 10^-3^ emerging roots cm^-2^ month^-1^ (**Figure 6B**).

**FIGURE 7:**
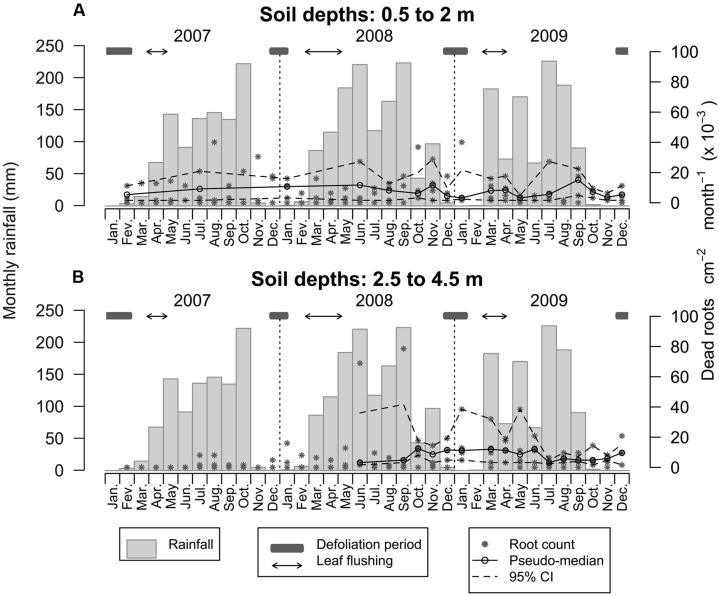
**Dynamics of root mortality over 3 years within the top 0.5–2.0m of the soil profile (A) and between 2.5 and 4.5 m (B).** Asterisks are emerging root counts determined in every root window at monthly intervals. Open circles represent the pseudo-median of dead root counts in the deeper root windows (*n* = 4 and *n* = 5 for the upper and lower plots, respectively) and dashed lines are 95% confidence intervals estimated using the Wilcoxon signed rank test.

The range of root mortality between soil depths of 0.5 and 2.0 m was 1.60 to 40.08 × 10^-3^ dead roots cm^2^ month, with an average of 6.33 × 10^-3^ emerging roots cm^-2^ month^-1^ (**Figure 7A**). Root mortality at these soil depths was relatively stable over time with the highest mortality rates observed from August to January. Below a depth of 2.0 m, the range of root mortality was 1.60–78.56 × 10^-3^ dead roots cm^-2^ month^-1^, with an average of 5.28 × 10^-3^ emerging roots cm^-2^ month^-1^ (**Figure 7B**). Following the initial period of root emergence at these soil depths, root mortality tended to remain at relatively high levels from June 2008 until June 2009 (a mean of 10.04 × 10^-3^ emerging roots cm^-2^ month^-1^), beyond which it stabilized at a lower level of 6.65 × 10^-3^ emerging roots cm^-2^ month^-1^.

Bivariate plots of monthly root length variations as a function of (1) average monthly rainfall, (2) monthly average of minimum daily soil temperature, and (3) average reference evapotranspiration are given in Supplementary Figure S2. There was a weak yet significant (as indicated by the low R-squared and *p*-values of the regressions) positive relationship between, on the one hand, average monthly rainfall and monthly root length variations (Supplementary Figure S2A) and on the other hand, monthly average of minimum daily soil temperature and monthly root length variations (Supplementary Figure S2C).

## Discussion

### Fine Root Emergence

We showed that root phenology differed along the soil profile and was not synchronized with rainfall patterns as we had hypothesized, particularly below a depth of 2.0 m. Within the first 2 m of the soil profile, the highest rates of root emergence occurred about 3 months after the onset of the rainy season, while deeper in the soil, root emergence remained low until the 11th month of the observation period and was not correlated with the rainfall pattern. Therefore, the shallow parts of the root system were more responsive to rainfall, as roots near the soil surface capture water from rainfall more readily than deeper roots. Deep roots only emerged once rainfall became scarcer and may reflect the need for trees to use increasingly deeper water resources during the dry season.

Below 2.0 m, the first peak of root emergence rates occurred in November and December 2007, followed by a period of high root emergence that spanned from July 2008 to January 2009 (with the maximum peak in January 2009). Surprisingly, the highest emergence rates below 2.0 m occurred during the period of aerial dormancy, i.e., with no leaves supplying resources for root growth through photosynthesis. Similar results, whereby broadleaf tree root growth occurs significantly during a period of aerial dormancy, were also found in a Mediterranean climate in *Juglans regia* L. (Germon et al., under revision). Abramoff and Finzi (2015) suggest that endogenous cuing (i.e., any factor that affects growth other than climate), and subsequent allocation of stored non-structural carbohydrates (NSC) are dominant drivers of root growth in subtropical and Mediterranean trees. Although the climate in our study was tropical, distinct rainy seasons are present, but soil and air temperatures remain warm, therefore water supply is likely the main limiting climatic factor, particularly in the upper soil horizons where less buffering exists against soil drying. In deeper soils, thermic and hydric buffering should thus allow for more constant rates of root growth throughout the year if endogenous cuing is not the main driver of growth. However, we found that the peak of deep root growth was delayed until late into the dry season. As tree root and stem NSC usually decline during the growing season and re-accumulate during aerial dormancy (Richardson et al., 2013), NSC re-accumulation rates may differ between shallow and deep roots, with a time lag resulting in delayed deep root growth. Nevertheless, as root emergence rates were so different between shallow and deep roots, it may be that the drivers between the two compartments are separate and distinct, with rainfall driving shallow root growth and endogenous cuing driving deep root growth. However, further studies using isotopes would be needed to support this hypothesis (Trumbore et al., 2006).

Between soil depths of 0.5 and 2.0 m, root mortality was relatively disconnected from variations in root emergence, although higher mortality values occurred toward the end of the rainy season, as did the highest emergence values. Below 2.0 m, from 2008 onward, peaks in root emergence and death were largely synchronized, e.g., in June and September 2008 as well as April–May 2009, suggesting the existence of a mechanism for the replacement of recently senesced roots. It is also possible that deep roots that first grew after the installation of the well, began to die, either because they could not be maintained by the tree (too costly in terms of resources) or because the relatively high rates of emergence during the second year were a response to the disturbance caused by the well installation, as often occurs in rhizotron experiments (Strand et al., 2008). Roots growing in the direction of the well may have had reduced access to resources (since the volume occupied by the well was inaccessible) thus suppressing root emergence (feedback response). Observed differences in root emergence could also be related to the presence/absence of roots in the immediate vicinity of observation windows, prior to their installation which increased the probability for early root emergence in windows near pre-existing roots. The total higher and lower rates of root emergence observed in 2008 and 2009, respectively, may also have been influenced by the total annual precipitation, which in 2009 (1002 mm) was only 79% of that in 2008 (1265 mm). The year 2007 was the driest during the observation period with only 965 mm total annual precipitation. However, the second semester of year 2009, was the driest second semester of the monitoring period with only 78% of the rainfall that had been monitored for the same period in the two preceding years. In the first 3 months of the rainy season 2009 (April–June) it rained less than 60% of that for the same period in 2008. However, the reduced emergence within the first 2 m, putatively related to drier conditions during the rainy season, was not counter-balanced by increased emergence at depth. Therefore, while our data support the hypothesis that deep root emergence might correspond to a safety net against water stress during the dry season, they do not point at the existence of a similar mechanism against dry periods occurring during the wet season.

The peak of evapotranspiration that occurs every year around March–April, was highest in 2007, intermediate in 2008 and lowest in 2009 (it did not occur in 2009 as high rainfall occurred as soon as March 2009). We hypothesize that high evaporative demand may be a signal that triggers root growth at the onset of the rainy season, particularly near the soil surface and the low evapotranspiration observed in 2009 may have resulted in a weaker pattern of root emergence in that year.

At all depths, in 2008, root emergence reached its lowest level throughout the period during which trees had already shed old leaves but not yet started to grow new leaves (i.e., February). Although this pattern could not be observed in the previous and following years, it might correspond to a dormancy state prior to the resumption of physiological activity with the first rains of the season.

### Fine Root Turnover

As generally reported in the literature (Chen and Brassard, 2013), we found that soil depth had a significant effect on root turnover, but rather unexpectedly, root turnover increased with soil depth from 0.73 yr^-1^ at 0.5 m to 2–3 yr^-1^ at greater depths, in sharp contrast with what is generally reported (Wu et al., 2013). However, most studies have been concerned with soil depth ranges that were much shallower than those in our study (Baddeley and Watson, 2005; Chen and Brassard, 2013). Similarly, we did not find any evidence that root radius had an influence on longevity, although this has also been reported in the literature (Baddeley and Watson, 2005; Chen and Brassard, 2013). Furthermore, we did not find a linear increase in root turnover with soil depth over the whole 4.5 m range investigated, which is consistent with the theory that several factors, both intrinsic and extrinsic, control fine root life span (Chen and Brassard, 2013). It is known that environmental parameters (e.g., temperature, water content, N availability, CO_2_ partial pressure) influence fine root turnover to variable degrees (Vogt et al., 1996). Therefore, we hypothesize that during dry periods, deeper distal roots underwent a physiological pruning process, whereby peripheral organs died, as also observed in shoots of *H. brasiliensis* (Chen and Cao, 2015).

### Fine Root Biomass and Carbon

Assuming that the RLD values that we obtained from soil coring are homogeneous over large volumes of soil, it can be inferred that fine root biomass below a depth of 1.0 m could account for more than half of the overall fine root biomass of the rubber trees measured [4.8 t ha^-1^ between 0.0 and 1.0 m compared to 5.8 t ha^-1^ between 1.0 and 4.0 m, with a mean SRL of ∼14 m/g^-1^ for roots ≤1 mm in diameter (Pierret et al., 2007a)]. As roots may also be present below a depth of 4 m (the bedrock was found at 7–8 m), total root biomass may be underestimated. Assuming that rubber tree root tissues have a mean organic carbon content of approximately 47% (Wauters et al., 2008), our results show that rubber tree roots ≤0.5 mm in diameter, on average account for about 5 t C ha^-1^. This value is similar, although slightly higher, than the 1.91–3.72 5 t C ha^-1^ range reported by Wauters et al. (2008) for coarser roots (2.5–25 mm in diameter) for a range of rubber tree clones from Western Ghana and Brazil. Similarly Cheng et al. (2007) estimated carbon stocks of 16.50 t C ha^-1^ for roots of all sizes, in rubber tree plantations at Hainan Island, China and Yuen et al. (2013) calculated total carbon stocks of the order of 4–32 t C ha^-1^ for rubber trees at six locations in Southeast Asia. Hence, the presence of fine roots at fairly low length densities over considerable soil depths can have important implications with regard to soil carbon accounting. As recently pointed out by Yuen et al. (2013), more attention should be given to sampling roots at appropriate depths if we are to improve baseline data on belowground carbon stocks. In addition, it should be acknowledged that there are still major uncertainties regarding (1) the reliability of coring versus imaging techniques for quantifying fine root biomass and turnover (Yuan and Chen, 2012) and (2) the way different fine root definitions might influence such quantifications (McCormack et al., 2015).

## Conclusion

We explored the interactions between fine root dynamics, the rainfall regime and soil along a 4.5 m profile using a root access-well. Our results reveal that root growth dynamics in the upper 2 m of soil surface were related mainly to precipitation patterns (Chairungsee et al., 2013). Deeper in the soil, root growth was more independent of rainfall and was likely driven by internal tree carbon allocation. We show that fine root production will impact soil carbon stocks and was higher than commonly reported (e.g., Brunner and Godbold, 2007), particularly at depth. Such an input of fine root related carbon in soils could be all the more significant considering the slow breakdown of fine roots in some sub-tropical tree species (Xiong et al., 2012). One major limitation of this work is that observations are taken from a single location, which means that inference and conclusions cannot be generalized. The results of this study thus advocate in favor of more field studies aimed at assessing precisely the production and fate of fine roots, not only near the soil surface but also very deep in the soil.

## Author Contributions

JLM and AP designed the experimental setup, implemented it in the field, analysed the data and wrote the paper. SG and SINA performed data collection in the field. CC, AS and SG have contributed significantly to the data analysis, discussing the results and writing of the paper.

## Conflict of Interest Statement

The authors declare that the research was conducted in the absence of any commercial or financial relationships that could be construed as a potential conflict of interest.

## Footnotes

1 www.gimp.org

2 http://rsbweb.nih.gov/ij/
