# Supplementary figures and images for: Seasonal Patterns of Fine Root Production and Turnover in a Mature Rubber Tree (Hevea brasiliensis Müll. Arg.) Stand- Differentiation with Soil Depth and Implications for Soil Carbon Stocks
Source: Front Plant Sci. 2015 Nov 27;6:1022. doi: 10.3389/fpls.2015.01022 (PMC4661276; doi:10.3389/fpls.2015.01022)

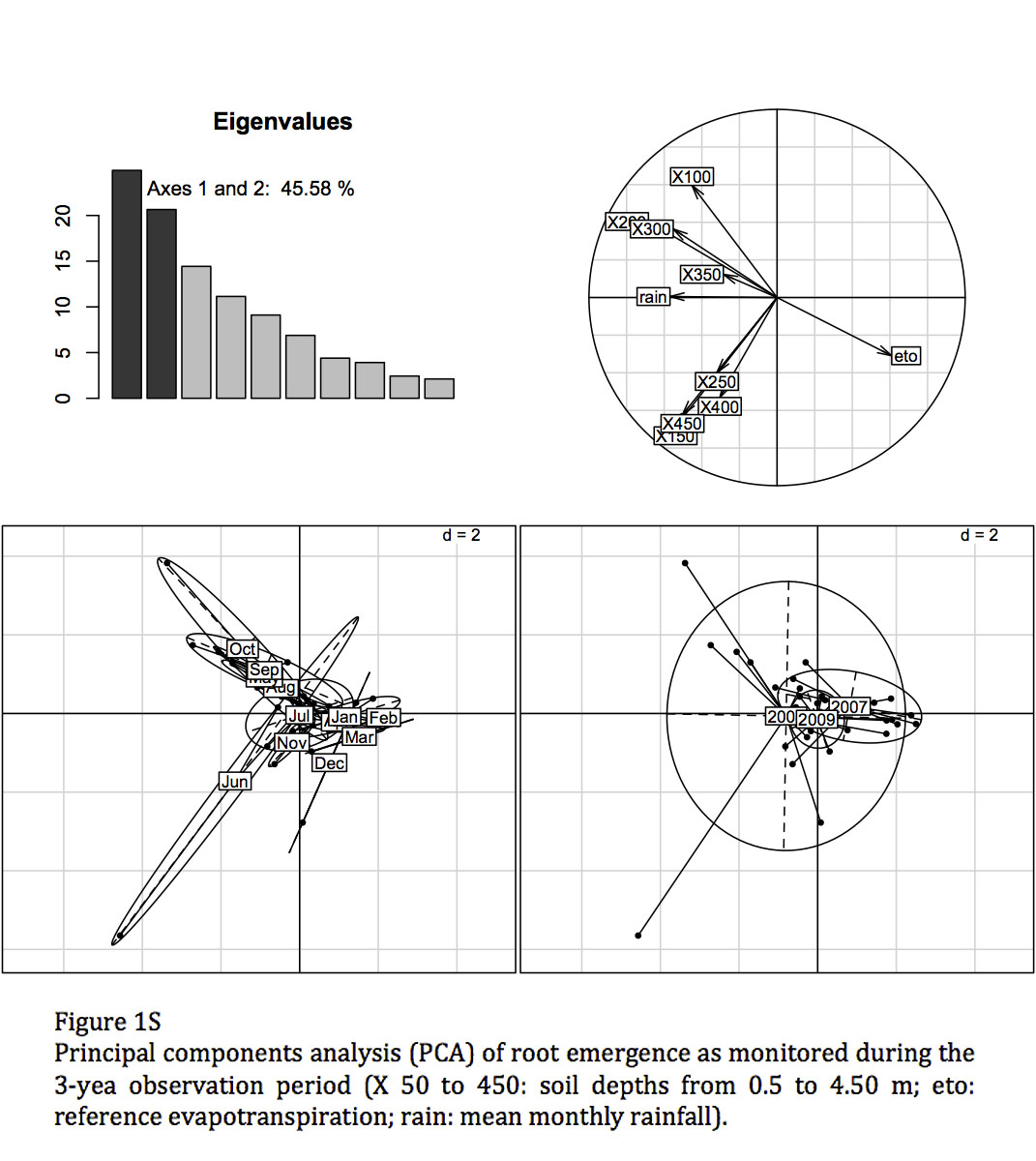

Supplement: Supplementary file 1 [file Image_1.JPEG]

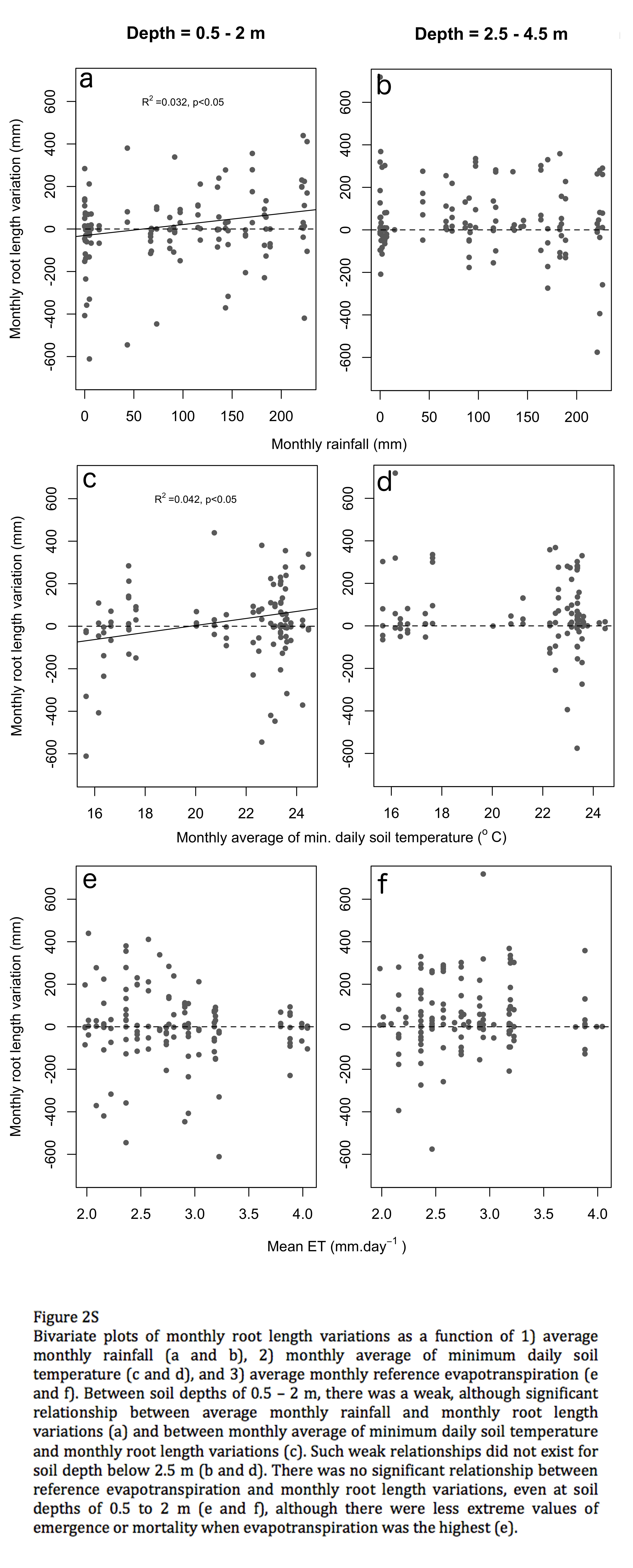

Supplement: Supplementary file 2 [file Image_2.JPEG]
